# Sonic hedgehog inhibitors prevent colitis-associated cancer via orchestrated mechanisms of IL-6/gp130 inhibition, 15-PGDH induction, Bcl-2 abrogation, and tumorsphere inhibition

**DOI:** 10.18632/oncotarget.6765

**Published:** 2015-12-26

**Authors:** Napapan Kangwan, Yoon-Jae Kim, Young Min Han, Migyeong Jeong, Jong-Min Park, Eun-Jin Go, Ki-Baik Hahm

**Affiliations:** ^1^ CHA Cancer Prevention Research Center, CHA Cancer Institute, CHA University, Seongnam, Korea; ^2^ Department of Gastroenterology, Gachon University Gil Medical Center, Incheon, Korea; ^3^ Digestive Disease Center, CHA University Bundang Medical Center, Seongnam, Korea

**Keywords:** sonic hedgehog (SHH), colitis associated cancer (CAC), anti-inflammation, 15-PGDH, cancer stem cell

## Abstract

Sonic hedgehog (SHH) signaling is essential in normal development of the gastrointestinal (GI) tract, whereas aberrantly activated SHH is implicated in GI cancers because it facilitates carcinogenesis by redirecting stem cells. Since colitis-associated cancer (CAC) is associated with inflammatory bowel diseases, in which SHH and IL-6 signaling, inflammation propagation, and cancer stem cell (CSC) activation have been implicated, we hypothesized that SHH inhibitors may prevent CAC by blocking the above SHH-related carcinogenic pathways. In the intestinal epithelial cells IEC-6 and colon cancer cells HCT-116, IL-6 expression and its signaling were assessed with SHH inhibitors and levels of other inflammatory mediators, proliferation, apoptosis, tumorsphere formation, and tumorigenesis were also measured. CAC was induced in C57BL/6 mice by administration of azoxymethane followed by dextran sodium sulfate administration. SHH inhibitors were administered by oral gavage and the mice were sacrificed at 16 weeks. TNF-α–stimulated IEC-6 cells exhibited increased levels of proinflammatory cytokines and enzymes, whereas SHH inhibitors suppressed TNF-α–induced inflammatory signaling, especially IL-6/IL-6R/gp130 signaling. SHH inhibitors significantly induced apoptosis, inhibited cell proliferation, suppressed tumorsphere formation, and reduced stemness factors. In the mouse model, SHH inhibitors significantly reduced tumor incidence and multiplicity, decreased the expression of IL-6, TNF-α, COX-2, STAT3, and NF-κB, and significantly induced apoptosis. In colosphere xenografts, SHH inhibitor significantly suppressed tumorigenesis by inhibiting tumorsphere formation. Taken together, our data suggest that administration of SHH inhibitors could be an effective strategy to prevent colitis-induced colorectal carcinogenesis, mainly by targeting IL-6 signaling, ablating CSCs, and suppressing oncogenic inflammation, achieving chemoquiescence ultimately.

## INTRODUCTION

Colorectal cancer (CRC) is one of the most commonly diagnosed malignancies and the third leading cause of cancer deaths in developed countries [1] Colitis-associated cancer (CAC) is the subtype of CRC that is associated with long-standing inflammatory bowel disorders (IBD) including Crohn's disease and ulcerative colitis, and accounts for 15–20% of CRC cases [2, 3]. Since IBD have clinical features of chronic relapsing inflammation, several proinflammatory cytokines such as TNF-α, IL-6, and IL-1β can induce mutagenic environments such as increased levels of intracellular reactive oxygen species and reactive nitrogen species, which induce DNA damage as well as epigenetic changes that silence tumor suppressors and promote tumor initiation [4–6]. These events link the pathogenesis of IBD and that of CAC. CAC can be preventable through regular surveillance of high-risk patients with IBD and intervention with potent anti-inflammatory agents. Unless CAC is detected early, patients have rather poor prognosis and show resistance to cancer therapeutics [7], which can be explained by the facts that cancer stem cell (CSC) foci still persist in inflamed colon tissue even after the removal of cancer tissues and the possible presence of other target implicated in oncogenic inflammatory mediators such as IL-6, regenerating gene-l alpha (REG-1α), *etc* beyond current targeting TNF-α [8].

The redox-sensitive NF-κB pathway, which regulates proinflammatory responses, is markedly activated in patients with IBD through transcriptional activation of the expression of several proinflammatory genes such as TNF-α, iNOS, IL-2, IL-6, IL-8, and IL-12. In particular, IL-6, a crucial NF-κB–dependent tumorigenic cytokine, binds to a soluble form of IL-6 receptor (IL-6R), and this complex interacts with gp130; the IL-6–IL-6R– gp130 complex dimerizes and induces phosphorylation of STAT3, a cytoplasmic protein that functions as a transcriptional activator and plays a pivotal role in the regulation of different types of immune and inflammatory responses. Phosphorylated STAT3 dimerizes and translocates into the nucleus, where it binds to specific DNA motifs and activates the transcription of distinct groups of genes implicated in inflammation and carcinogenesis [9]. The importance of the IL-6 family of proinflammatory cytokines and STAT3 in CAC is well known [10].

Chronic inflammatory diseases of the gut, including IBD, are accompanied by increased epithelial sonic hedgehog (SHH) signaling, the abnormal persistence of which or ectopic expression of SHH may stimulate abnormal stem cell hyperplasia, providing a critical step in the neoplastic transformation of the gut tissues [11]. Conversely, inhibition of SHH signaling reduces local neutrophil infiltration and expression of proinflammatory cytokines such as TNF-α and IL-6 [12]. Re-activation of embryonic SHH signaling, which is essential in normal GI tract organogenesis, can lead to an abnormal healing process associated with oncogenesis, cancer resistance to therapy, and relapse [13, 14]. Among these stem cell pathways, the SHH signaling pathway is essential for prenatal development and controlling differentiation, maintaining stem cell niches, and determining the cellular response to injury in adults.

In the current study, we documented the role of IL-6/IL-6R/gp130/STAT3 signaling in CAC, investigated whether SHH inhibitors can abrogate oncogenic IL-6 signaling, impose anti-inflammatory effects, preserve 15-hydroxyprostaglandin dehydrogenase (15-PGDH as confidential tumor-suppressive pathways, induce apoptosis through *Gli*-*1*–dependent down-regulation of Bcl-2, and kill CSC presented in colosphere xenografts.

## RESULTS

### SHH inhibitors significantly inhibit TNF-α–stimulated IL-6 and its receptor IL-6R/gp130 signaling through STAT3

**Figure 1:**
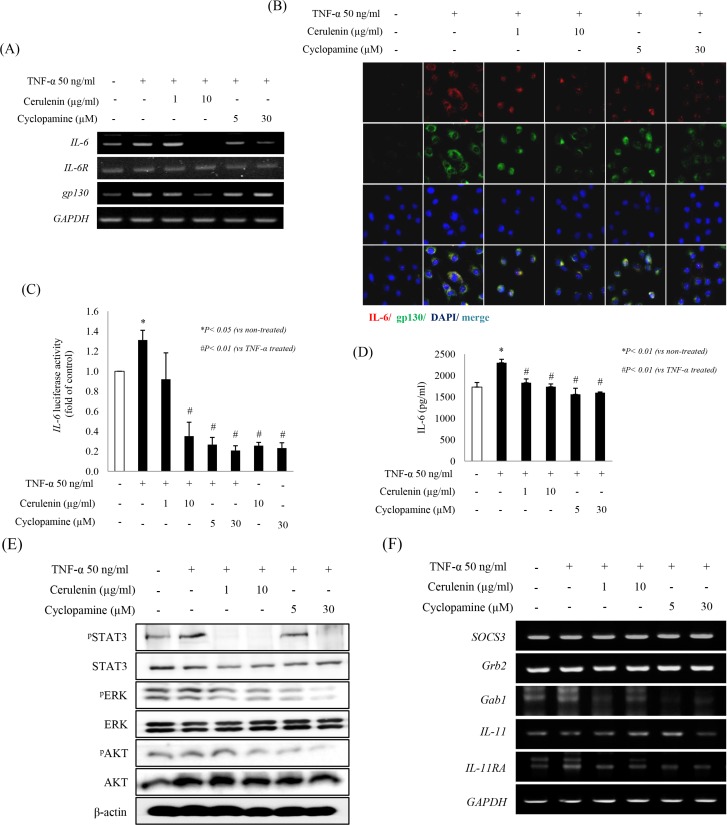
Blocking the SHH pathway suppresses the IL-6/STAT3 pathway *in vitro* (**A**) IEC-6 cells were pretreated with SHH inhibitors for 1 h and then stimulated with 50 ng/ml TNF-α for 6 h. Expression of mRNAs for IL-6 and its receptors was quantified by RT-PCR and expressed relative to that of GAPDH. SHH inhibitors decreased the levels of TNF-α-induced IL-6 and mRNAs for its receptors *IL-6R* and *gp130*. (**B**) IL-6 (red) and gp130 (green) were detected by multiple immunofluorescence labeling and confocal laser scanning microscopy (×400). Sections were counterstained with DAPI (blue). The bottom panel shows a merged image. (**C**) IEC-6 cells were transiently transfected with luciferase reporter plasmid containing IL-6 promoter elements. After 24 h, cells were pretreated with SHH inhibitors for 1 h, and then stimulated with 50 ng/ml TNF-α for 6 h. Luciferase activity was then determined and normalized to β-galactosidase activity to account for differences in transfection efficiency. (**D**) IL-6 concentration in conditioned medium was analyzed by ELISA. (**E**) Western blot analysis showed the effect of treatment with SHH inhibitors for 3 h on the levels of TNF-α–induced STAT3, ERK, and Akt. β-actin was used as an equal loading control. (**F**) Regulation of STAT3-responsive genes including *SOCS3, Grb2*, and *Gab1* was determined by RT-PCR. All data are representative of at least three independent experiments.

The IL-6/STAT3 signaling pathway has an essential role in driving a variety of biological responses to inflammatory challenge and is the principal signaling pathway implicated in CAC [15]. To study the effects of the SHH inhibitors cerulenin and cyclopamine on IL-6/gp130/STAT3 signaling, IEC-6 cells were challenged with 50 ng/ml TNF-α for 6 h (Supplementary Figure 1A). TNF-α significantly increased the levels of IL-6 and gp30, whereas treatment with 10 mg/ml cerulenin or 30 μM cyclopamine significantly decreased the levels of IL-6 or gp130 mRNAs (Figure 1A) and proteins (Figure 1B). Cyclopamine and cerulenin significantly inhibited TNF-α–induced *Cox-2*, *IL-6*, and *Smo* (Supplementary Figure 1B). Therefore, we next checked the effects of SHH inhibitors on the activity of luciferase expressed under the control of the IL-6 promoter. IL-6 promoter activity was significantly increased by TNF-α, and its TNF-α–induced activity was significantly decreased by cerulenin or cyclopamine (*p* < 0.01 for both inhibitors, Figure 1C). To verify these findings, the level and location of IL-6 and gp130 receptor were determined by ELISA (Figure 1D) and confocal imaging (Figure 1B), respectively. TNF-α significantly increased, whereas SHH inhibitors significantly decreased the levels of IL-6 and gp130. Since IL-6 signaling is regulated by STAT3 activation, we further examined changes in STAT3 phosphorylation after treatment with SHH inhibitors. As shown in Figure 1E, 1 or 10 mg/ml cerulenin or 30 μM of cyclopamine significantly inactivated STAT3. Although ERK and AKT inactivation was also noted in the presence of SHH inhibitors, these kinases were not significantly activated upon TNF-α stimulation of IEC-6 cells (in colon cancer cells HCT-116, ERK and AKT were significantly activated by TNF-α and significantly inactivated by SHH inhibitors; data not shown). The levels of *Gab1* and *IL-11RA* mRNAs were significantly increased by TNF-α in IEC-6 cells, whereas SHH inhibitors significantly reduced the expression of these genes (Figure 1F).

### SHH inhibitors suppress TNF-α–induced inflammatory mediators by inactivating NF-κB signaling

**Figure 2:**
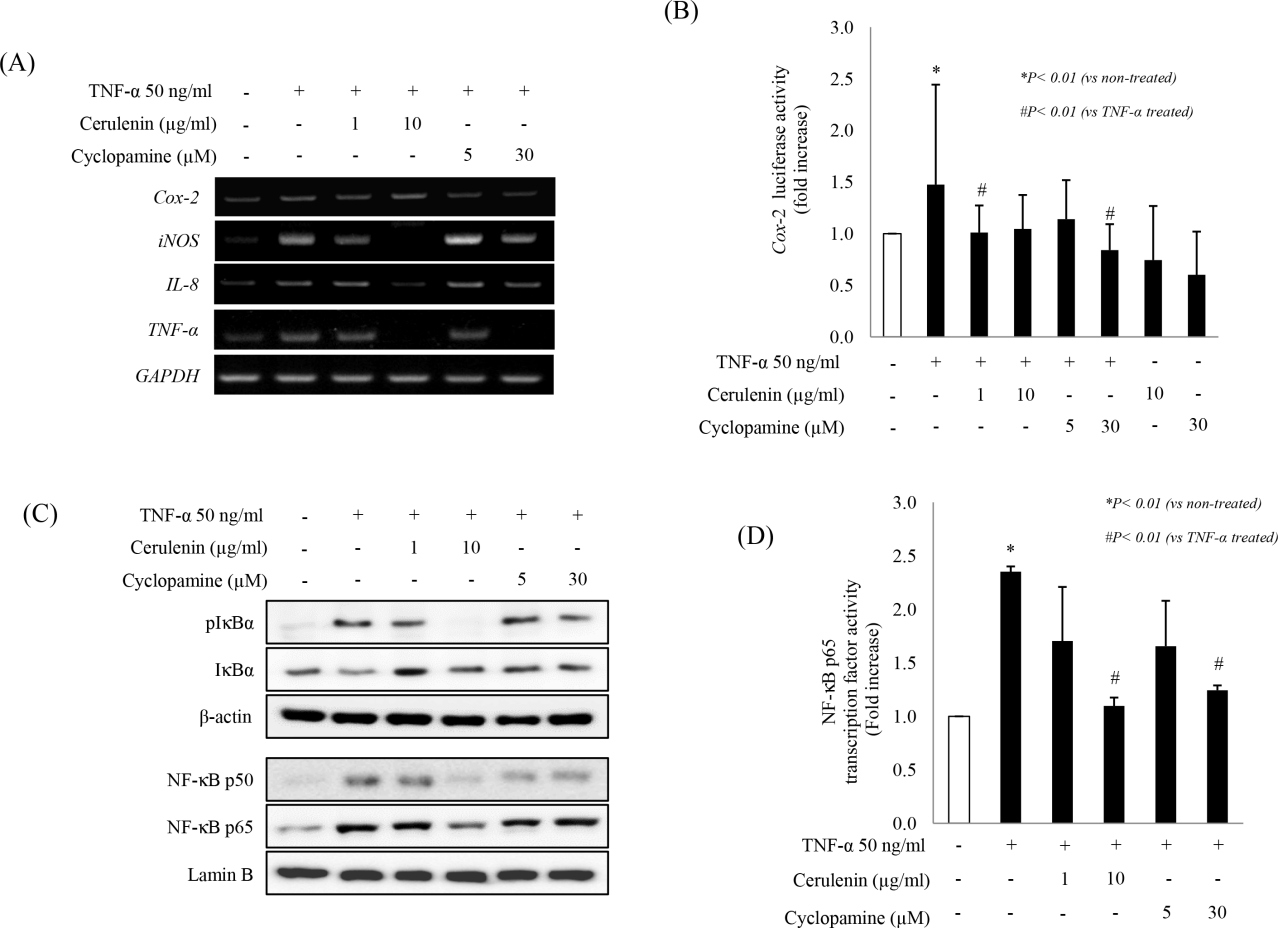
SHH inhibitor inhibits TNF-α–induced proinflammatory cytokines and inactivates NF-κB transcription factor (**A**) Changes in the expression of mRNAs for proinflammatory cytokines and enzymes including COX-2, TNF-α, iNOS, IL-8, and MCP-1 were evaluated by RT-PCR. IEC-6 cells were pretreated with the indicated concentrations of SHH inhibitors for 1 h, and then stimulated with 50 ng/mL of TNF-α for 6 h. (**B**) IEC-6 cells were transiently transfected with luciferase reporter plasmid containing *Cox*-*2* promoter elements. After 24 h, cells were pretreated with SHH inhibitors for 1 h, and then stimulated with 50 ng/ml TNF-α for 6 h. Luciferase activity was then determined and normalized to β-galactosidase activity to account for differences in transfection efficiency. Three independent assays were performed and the data shown are the mean ± SD. (**C**) Effect of SHH inhibitors on TNF-α–induced phosphorylation of IκBα. IEC-6 cells were pretreated with SHH inhibitors for 1 h followed by 50 ng/mL of TNF-α for 3 h. Nuclear localization of NF-κB was determined by Western blot analysis. (**D**) Transcription factor activity of NF-kB-p65 was analyzed by ELISA.

To elucidate the effects of SHH inhibitors on additional TNF-α–induced inflammatory mediators, we measured *Cox-2*, *TNF-α*, *iNOS*, and *IL-8* expression (these inflammatory mediators are all known to be involved in colitis and CAC [16]) and found their significant down-regulation in the presence of 10 mg/ml cerulenin or 30 μM cyclopamine (Figure 2A). Cerulenin or cyclopamine also significantly reduced *Cox-2* promoter activity assessed by using a luciferase assay (*p* < 0.01; Figure 2B). Since transcription of these proinflammatory genes is tightly regulated by the transcription factor NF-κB [17], we measured the levels of phosphorylated IκBα in the cytoplasmic fraction and NF-κB in the nuclear fraction. Both cerulenin (10 mg/ml) and cyclopamine (30 μM) significantly inactivated the NF-κB pathway (Figure 2C). Similarly, both 10 mg/ml cerulenin and 30 μM cyclopamine significantly inhibited NF-κB transcriptional activity (*p* < 0.01, Figure 2D).

### SHH inhibitors reduce cell survival, induce apoptosis and inhibit cell cycle

**Figure 3:**
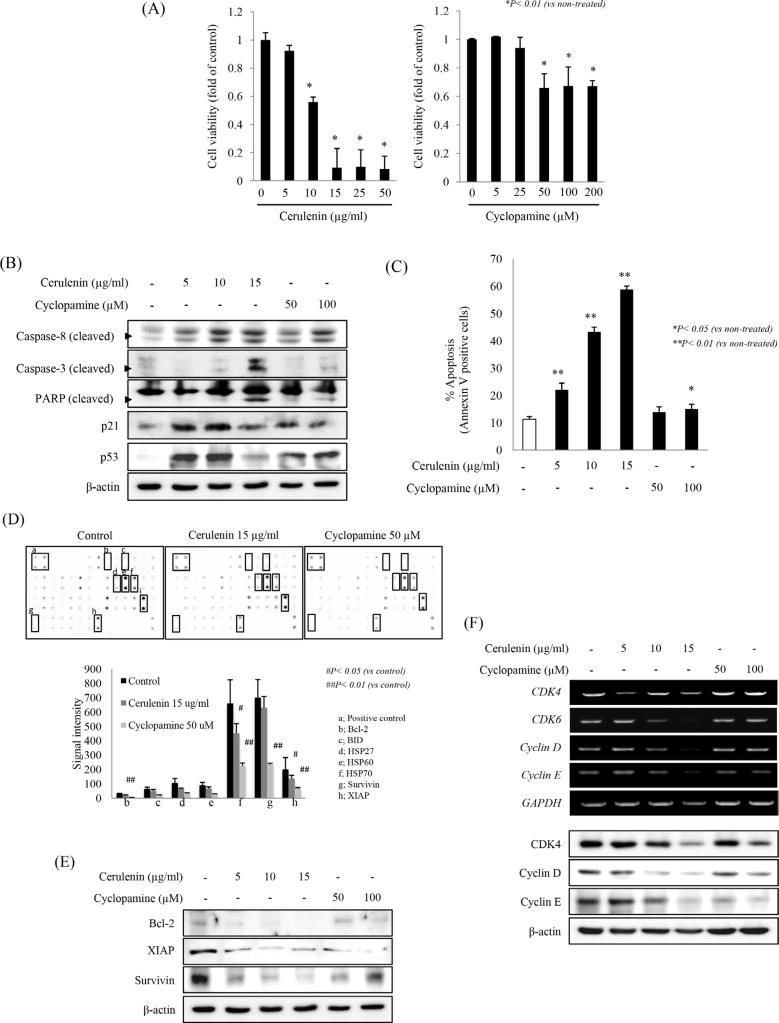
SHH inhibitors provoke apoptosis and a change in cell cycle in HCT-116 cells (**A**) HCT-116 cells were treated with the indicated concentrations of SHH inhibitors for 24 h, and their viability was determined by the MTT assay. The results are presented relative to the viability of untreated cells (control), which was considered as 1, and are means ± SD. *p < 0.01 in comparison with untreated cells. (**B**) Western blots showing a significant increase in apoptotic proteins (cleaved caspase-3, cleaved caspase-8, and cleaved PARP) after treatment with SHH inhibitors for 24 h. (**C**) HCT-116 cells were treated with SHH inhibitors for 24 h and analyzed by flow cytometry with FITC–Annexin V and propidium iodide (PI) staining to verify the induction of apoptosis. (**D**) Apoptosis protein array analysis of HCT-116 cells (untreated or treated with 15 μg/ml cerulenin or 50 μM cyclopamine for 24 h). (**E**) Western blots showing a significant decrease in anti-apoptotic proteins (Bcl-2, survivin, and XIAP). (**F**) The expression of the cell cycle markers CDK4, Cyclin D1, Cyclin E, and p21 was assessed by Western blot analysis after treatment with an SHH inhibitor. Data are means ± SD, *p < 0.05, **p < 0.01 in comparison with untreated control.

To investigate the effects of SHH inhibitors on cell survival and proliferation, we first used MTT assays to measure cell viability after treatment with each SHH inhibitor for 24 h. There was a significant and concentration-dependent decrease in cell viability after incubation with 10 to 50 μg/ml cerulenin and a significant decrease in cell viability after incubation with 50 to 200 μM cyclopamine (*p* < 0.01, Figure 3A). Flow cytometry showed a similar outcome (Supplementary Figure 2A). To see whether reduced cell viability was caused by apoptosis induction, the levels of caspase-8, caspase-3, cleaved PARP, p21, and p53 were examined by Western blotting. Significant cleavage of PARP, caspase-8, and caspase-3 was noted after incubation with SHH inhibitors, in line with significant induction of p21 and p53 (Figure 3B). Flow cytometry showed that these pro-apoptotic events were more prominent with cerulenin administration and were dose dependent (*p* < 0.01, Figure 3C). To further evaluate the effects of SHH inhibitors on the execution of apoptosis, we treated HCT-116 cells with cerulenin (15 μg/ml) or cyclopamine (50 μM), lysed them and screened the apoptotic markers using an apoptotic protein array (Figure 3D, Supplementary Figure 2B). The anti-apoptotic protein Bcl-2 and the pro-apoptotic protein BID protein were significantly down-regulated. The treatment also reduced the levels of the inhibitors of apoptosis XIAP and survivin. The anti-apoptotic heat shock proteins (HSPs) HSP60, HSP70, and HSP27, which are a result of oxidative stress in the cell, were also significantly repressed (*p* < 0.01). The results of the protein array were validated by Western blotting for Bcl-2, XIAP, and survivin (Figure 3E). To further validate, we used multiplex qRT-PCR analyses of cyclin D1, cyclin E, CDK4, and p21 (Supplementary Figure 2C). We found that SHH inhibitors reduced the levels of cyclin D1 and cyclin E proteins (Figure 3F). A DCFH-DA assay showed that SHH inhibitors induced oxidative stress in cells (Supplementary Figure 2D). To confirm the effect SHH inhibitors on the SHH signaling pathway in colorectal cancer, we investigated their effects on proliferation and SHH/Patched/Smo/Gli signaling in HT-29 colorectal cancer cells. We observed a significant and concentration-dependent decrease in cell viability after 24 h of incubation with 10 to 50 μg/ml cerulenin and a significant decrease in cell viability in the presence of 25 to 200 μM cyclopamine (*p* < 0.001, Supplementary Figure 3A). Cerulenin and cyclopamine significantly decreased the levels of the SHH, Patched, Smo, and Gli-1 proteins (Supplementary Figure 3B) and corresponding mRNAs (Supplementary Figure 3C).

### SHH inhibitors suppress colonocyte migration and proliferation

**Figure 4:**
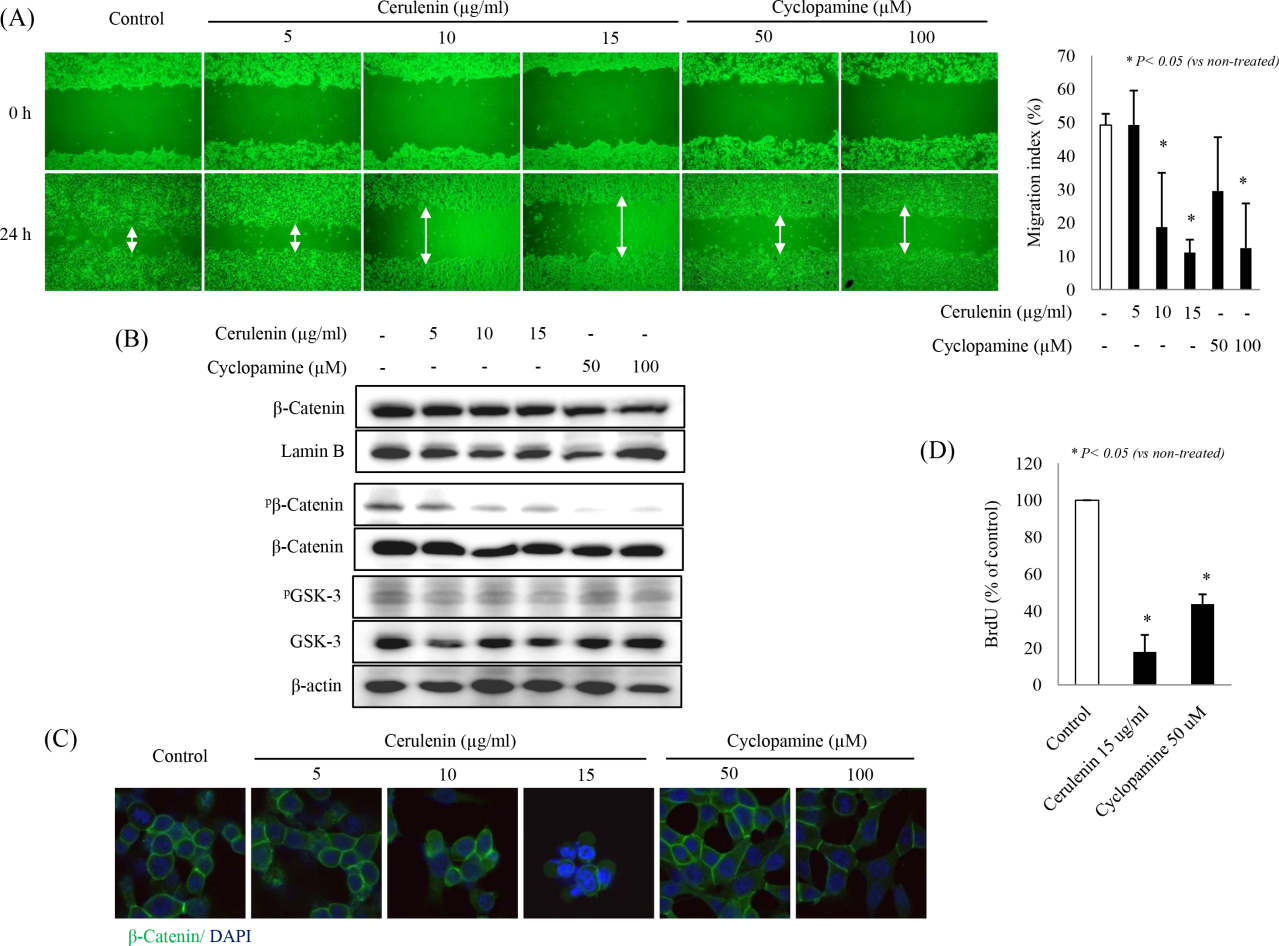
SHH inhibitors suppress HCT-116 cell migration and proliferation (**A**) Wound healing assays were carried out in 6-well plates. A line of cells in a confluent monolayer was damaged by scratching. Cell motility was examined under a light microscope (×40 magnification) for the indicated durations (24 h). (**B**) SHH inhibitor treatment decreased phosphorylation of β-catenin, but did not change the levels of GSK-3 (**C**) β-Catenin expression in different animal groups was assessed by using immunofluorescence staining. (**D**) The percentage of BrdU-positive cells was significantly lower in the presence of SHH inhibitors than in the control group.

Next, we conducted a wound healing assay to check the effects of SHH inhibitors on proliferation and migration of HCT-116 cells. A significant inhibition of wound healing was observed with both cerulenin and cyclopamine (*p* < 0.05, Figure 4A), in contrast to a nearly complete wound closure in control cells. Since β-catenin activation is known to be the principal driver of cell proliferation and migration, we checked β-catenin nuclear translocation using nuclear fractions and its phosphorylation levels using cytoplasmic fractions. Cerulenin or cyclopamine significantly reduced β-catenin phosphorylation and nuclear translocation (Figure 4B and 4C). Likewise, SHH inhibitors also decreased the number of BrdU-positive cells (*p* < 0.05, Figure 4D). All these findings are consistent with a suggestion that SHH inhibitors significantly inhibit cell proliferation and migration.

### SHH inhibitors significantly inhibit the expression of stemness factors as well as tumorsphere formation and survival

**Figure 5:**
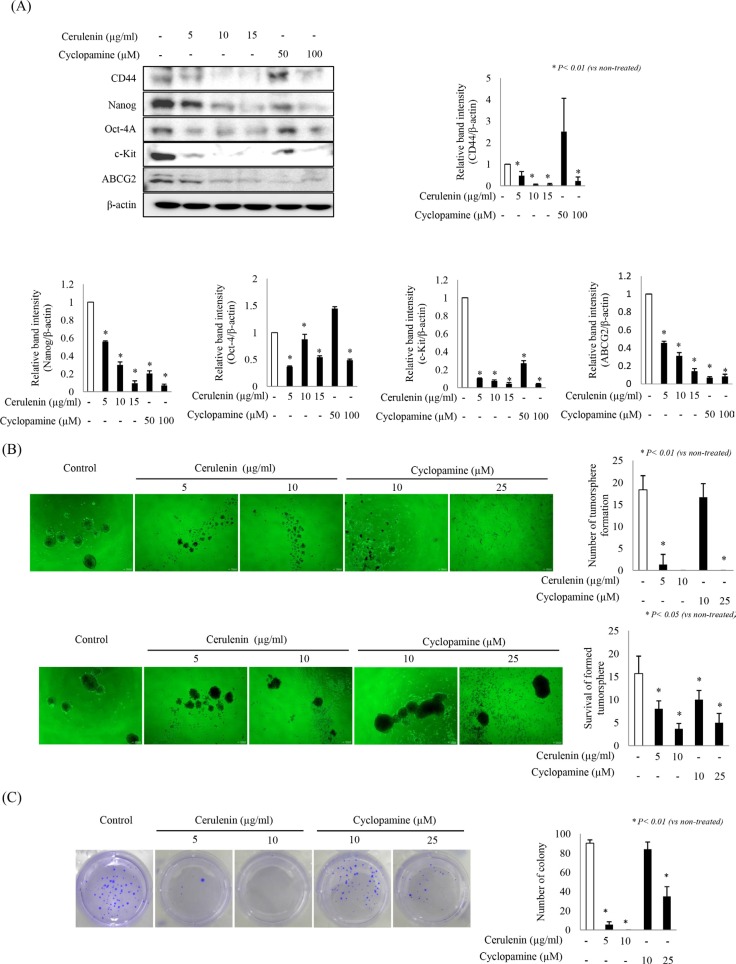
Effects of SHH inhibitors on stemness and sphere formation (**A**) Blocking the SHH pathway inhibited the expression of the colon cancer stem cell markers CD44, Nanog, Oct-4A, c-Kit, and ABCG2. HCT-116 cells were treated with SHH inhibitors for 24 h and cell extracts were analyzed by Western blotting. (**B**) SHH inhibitors attenuated tumorsphere formation and survival in a dose-dependent manner (upper part). Secondary tumorspheres were grown in serum-free medium (as described in Methods) for 7 days in the presence or absence of SHH inhibitors. The total number of tumorspheres formed from 500 single cells is shown in the lower part. (**C**) Effects of SHH inhibitors on the colony formation ability of tumorsphere cells under anchorage-independent conditions were assessed by soft agar assays. A representative photograph of a colony is shown on the left. Statistical analysis is shown on the right. All data are representative of three independent experiments.

Stem cell pathways may play an important role in colon cancers and may be potential treatment targets. Among these pathways, the SHH signaling pathway is essential in either prenatal development in embryonal cells or in controlling differentiation, maintaining stem cell niches, and determining the cellular response to injury in adults [18]. Treatment with SHH inhibitors for 24 h significantly reduced the expression of all stemness markers examined (CD44, Nanog, Oct-4A, c-kit, and ABCG2) in a dose-dependent manner (*p* < 0.01; Figure 5A). Based on these findings, we hypothesized that SHH inhibitors can inhibit tumorsphere formation or ablate tumorspheres. Indeed, various concentrations of SHH inhibitors (except 10 μM cyclopamine) significantly inhibited tumorsphere formation compared to control (*p* < 0.01, Figure 5B, upper part). Addition of cerulenin or cyclopamine after tumorsphere formation significantly reduced survival of colospheres in a dose-dependent manner (*p* < 0.05, Figure 5B, lower part). Using an anchorage-independent growth assay, we further evaluated whether SHH inhibitors would affect the colony-forming capacity of HCT-116 tumorsphere cells. As shown in Figure 5C, the colony-forming capacity was significantly reduced in the presence of SHH inhibitors in a dose-dependent manner (*p* < 0.01). These results suggest that SHH inhibitors significantly reduce either tumorsphere formation or tumorsphere survival in a dose-dependent manner.

### SHH inhibitor significantly prevented azoxymethane-initiated, dextran sodium sulfate–promoted CAC in mice

**Figure 6:**
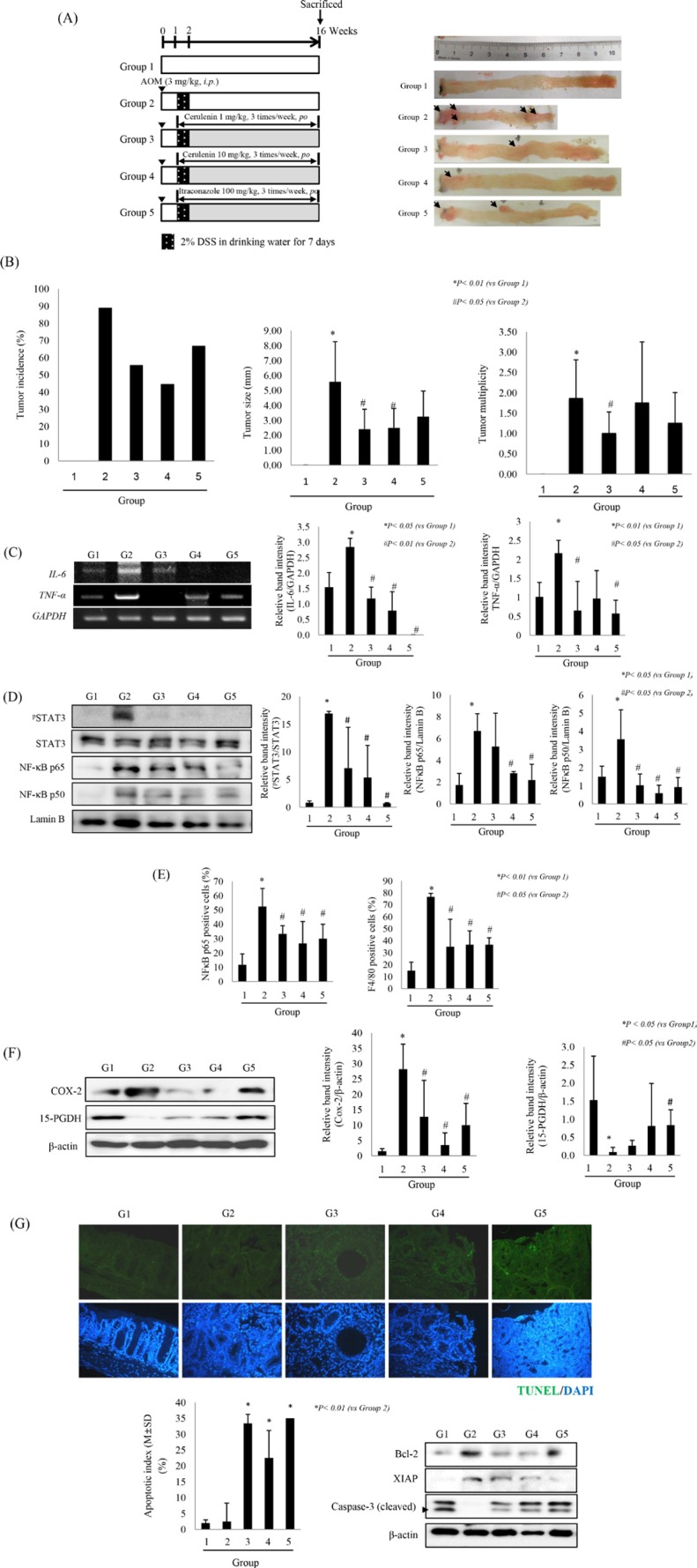
SHH inhibitors prevent AOM-initiated, DSS-promoted colitis-associated cancer (**A**) Overview of the experimental protocol of the AOM+DSS model (left panel) and representative gross appearance of mouse colons (right panel). Group 1; control, Group 2; AOM + DSS, Group 3; AOM + DSS + cerulenin 10 mg/kg, Group 4; AOM + DSS + cerulenin 30 mg/kg, Group 5; AOM + DSS + itraconazole 100 mg/kg. Black arrows indicate tumor location in colon tissue (right panel). (**B**) Tumor incidence (left), multiplicity (middle), and size (right). Data are means ± SD (*n* = 9 in each group). Inhibition of the SHH pathway decreased the expression of proinflammatory cytokines, inactivated the NF-κB and STAT3 pathways and promoted apoptosis in a colitis-associated colon cancer mouse model. (**C**) The levels of IL-6 and TNF-α mRNAs were determined by RT-PCR. (**D**) SHH inhibitors inactivated NF-κB and STAT3. Nuclear extracts were analyzed by Western blotting (*n* = 4). (**E**) Localization and expression levels of NF-κB and F4/80 were analyzed by immunohistochemistry in different groups (see Supplementary Figure 5A and 5B for immunohistochemical staining of NF-κB p65 and F4/80, respectively). (**F**) Effect of SHH inhibitors on the expression of COX-2 and 15-PGDH. The mean expression level is shown for each group. (**G**) TUNEL staining was performed to compare apoptosis in different groups (×200 magnification). Apoptotic index is shown for each group. *p < 0.01 *vs.* Group 2 (lower left). Equal amounts of total protein extracted from colon tissues were subjected to Western blot analysis using antibodies against Bcl-2, XIAP, and cleaved caspase-3. Data are means ± SD (lower right).

To validate the above cancer-preventive actions of SHH inhibitors *in vivo*, we used the azoxymethane (AOM)-initiated, dextran sodium sulfate (DSS)-promoted CAC model (Figure 6A). Mice were sacrificed on the 16th week; they showed a significant reduction in colon length and in the number of developed colitic tumors (arrows in Figure 6A). When we analyzed tumor incidence, tumor multiplicity, and tumor size, the cerulenin-treated groups (3 and 4), showed a significant decrease in tumor incidence, tumor multiplicity and tumor size (*p* < 0.05, Figure 6B). Since our *in vitro* experiments described above consistently showed that SHH inhibitors inhibit IL-6 and TNF-α signaling, we measured the expression of IL-6 and TNF-α in each group. A significant reduction in the levels of IL-6 and TNF-α was noted in SHH inhibitor–treated groups in comparison with the control group (*p* < 0.01, Figure 6C). We also measured STAT3 phosphorylation and NF-κB nuclear translocation and found that both were significantly inhibited by SHH inhibitor (*p* < 0.05, Figure 6D). Serum levels of IL-6 and TNF-α were also significantly decreased by SHH inhibitors (Supplementary Figure 4A and 4B). Because transcriptional activation of these cytokines were all provoked with chemotaxis including macrophages and monocytes [19], we carried out immunohistochemical staining with antibodies against NF-κB p65 and anti-F4/80. The numbers of cells expressing NF-kB p65 or F4/80 were significantly increased in the AOM+DSS group compared with the normal group (*p* < 0.01, Supplementary Figure 4C and 4D; Figure 6E), but was significantly decreased in the presence of SHH inhibitors (*p* < 0.05, Figure 6E). Interestingly, the expression of COX-2 was significantly increased in the AOM+DSS group (*p* < 0.05, Figure 6F), whereas that of 15-PGDH, a prostaglandin-degrading enzyme that is highly expressed in normal colon mucosa but is ubiquitously lost in human colon cancers [20], was significantly decreased in the AOM+DSS group (*p* < 0.05, Figure 6F). SHH inhibitor significantly decreased COX-2 expression and significantly increased that of 15-PGDH in comparison with the AOM+DSS group (*p* < 0.05, Figure 6F). Since our *in vitro* experiments described above, SHH inhibitors significantly induced apoptosis executors and significantly attenuated anti-apoptotic molecules, we performed TUNEL staining and Western blot analysis of cleaved caspase-3, XIAP, and Bcl-2 in different mouse groups. As shown in Figure 6G, a significant increase in the apoptotic index and in the level of cleaved caspase-3 was observed in the group treated with SHH inhibitors. These results suggest that oral administration of SHH inhibitors provoked significant apoptosis in CAC.

### Significant inhibition of colosphere formation and tumor xenograft by SHH inhibitors

**Figure 7:**
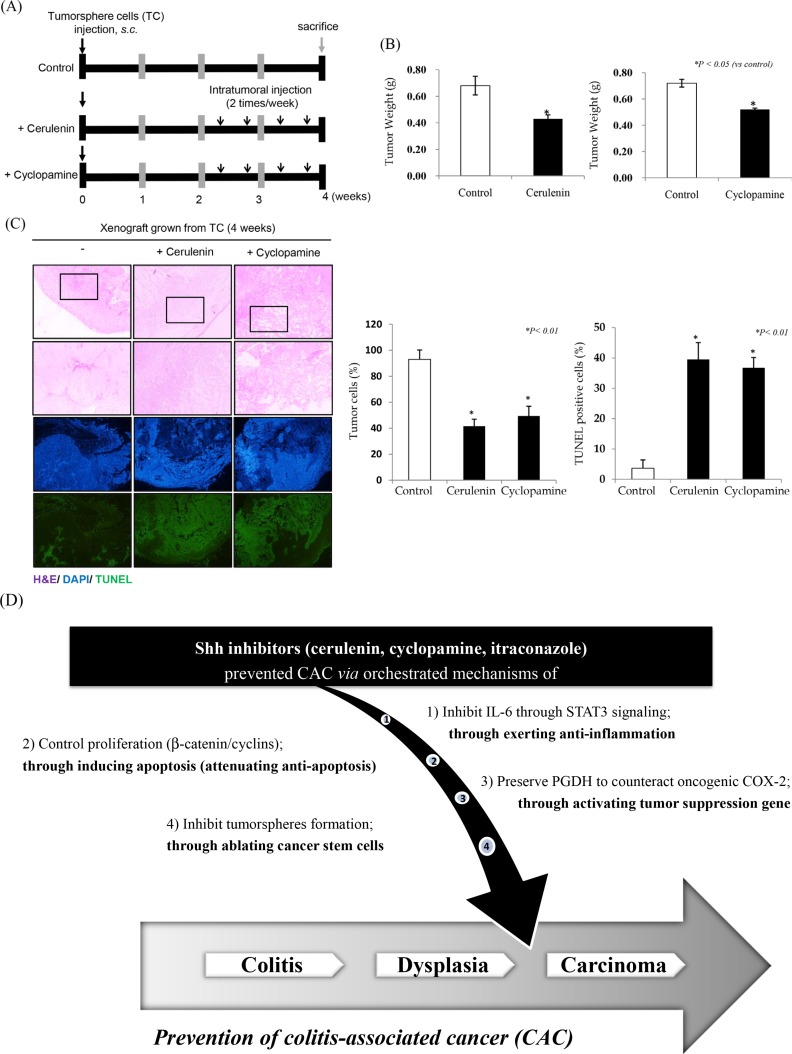
SHH inhibitors significantly inhibit cancer stem cell activation (**A**) The overall scheme of the use of SHH inhibitors to prevent colitis-associated colon cancer. (**B**) Mean tumor weights in the control group and groups treated with SHH inhibitors (cerulenin or cyclopamine). Both SHH inhibitors significantly inhibited tumorigenesis (*p* < 0.05). (**C**) Xenograft tumors were subjected to H & E, DAPI, and TUNEL assay. The SHH inhibitors significantly decreased the numbers of tumor nests and living tumor cells, and significantly increased the number of TUNEL-positive cells, *p < 0.01 (×100 magnification). (**D**) A scheme of the preventive actions of SHH inhibitors against colitis-associated cancer: 1) significant inhibition of IL-6 signaling through STAT3 inactivation as well as TNF-α attenuation through NF-κB repression, 2) significant inhibition of β-catenin–dependent cell proliferation and significant induction of apoptosis, 3) preservation of 15-hydroxyprostaglandin dehydrogenase (15-PGDH) to counteract oncogenic signaling of COX-2, and 4) ablation of cancer stem cells resulting in significant inhibition of tumorsphere formation.

Since SHH signaling has been implicated in embryogenesis, SHH inhibitors can specifically target CSCs [21]. Therefore, we hypothesized that SHH inhibitors would be able to ablate CSCs. Figure 5B and 5C shows that SHH inhibitors specifically and significantly inhibited colosphere formation, supporting the hypothesis that their cancer-preventive effects might be due to CSC ablation (Figure 7A). SHH inhibitors significantly inhibited tumorigenesis in a tumorsphere xenograft model (*p* < 0.05, Figure 7B). Compared to the colon cancer cell xenograft model, colospheres induced significant faster tumorigenesis and larger tumors (data not shown), whereas SHH inhibitors significantly inhibited tumorigenesis. Even more remarkable changes were noted on pathological observation of resected tumors: besides significantly decreased tumor size, the presence of intact tumor cells in tumor nests was significantly decreased in the group treated with SHH inhibitors (*p* < 0.01, Figure 7C). The fact that intact tumor sheets were significantly decreased by SHH inhibitors in addition to a significant reduction in tumor size was further confirmed by TUNEL staining of resected xenograft tumors: the number of TUNEL-positive cells was significantly increased by SHH inhibitors (*p* < 0.01, Figure 7C).

## DISCUSSION

In the current study, we documented, for the first time, that SHH inhibitors can prevent CAC through the following novel mechanisms: 1) significant inhibition of IL-6 signaling through STAT3 inactivation as well as TNF-α attenuation through NF-κB repression, 2) significant inhibition of β-catenin–dependent cell proliferation accompanied by significant induction of apoptosis, 3) preservation of 15-PGDH to counteract oncogenic signaling of COX-2, and 4) ablation of CSCs as shown by significant inhibition of tumorsphere formation; all these concerted mechanisms are schematically summarized in Figure 7D. Chronic inflammation has been recognized as an important risk factor for CRC, because it can promote carcinogenesis by inducing gene mutations, inhibiting apoptosis, or stimulating angiogenesis and cell proliferation [6]. Especially in CAC, gene mutations could occur in both neoplastic and normal cells, and were attributed to oxidative stress to DNA caused by chronic inflammation. In this respect, NF-κB provides a mechanistic link between inflammation and cancer, whereas other factors such as TNF-a and IL-6–induced signaling have been recently shown to promote tumor growth in colitic carcinogenesis [22]. The proinflammatory mediators, especially cytokines and chemokines, which function as driving forces for carcinogenesis, participate in every stage of initiation, promotion, and progression implicated in carcinogenesis [23]. Therefore, chemopreventive compounds inhibit carcinogenesis by inhibiting cell proliferation, inducing apoptosis, and suppressing the proinflammatory cytokines in adenocarcinomas that develop in the inflamed colon [24, 25]. Although TNF-α inhibition has been acknowledged as a potential strategy to prevent CAC, our study suggests that SHH inhibition to target IL-6 is another strategy to either prevent cancer or kill CSCs, which was never achieved before the current investigation.

SHH signaling is associated with processes such as gut development and maintenance of gut homeostasis [26, 27]. For instance, the SHH protein is frequently detected in gastric metaplasias in the adult human GI tract. Since it has recently been reported that epithelial malignancies of the adult gut are induced by both chronic inflammation and abnormal SHH expression or signaling, and that inflammatory environments often increase the expression of SHH ligands [28, 29], the SHH signaling–related transcription factor *Gli*-1 is a central mediator of the IL-6 signaling network and thereby can initiate the tumor microenvironment in CAC and regulate the progression of precursor lesions and tumor formation. Inversely, the loss of *Gli*-1 function diminishes IL-6 signaling from tumor-associated fibroblasts, which prevents progression of pancreatic precursor lesions to advanced stages [30]. In the current study, we demonstrated, for the first time, that the use of SHH inhibitors can be an effective strategy to prevent inflammation-induced carcinogenesis, especially CAC, from efficient inhibition of IL-6 and its signaling to global inhibition of several inflammatory mediators (Figure 1). Although we have reported previously that administration of anti-inflammatory agents such as infliximab, 8-OHdG, and pantoprazole effectively prevented CAC mainly by inhibiting TNF-α and related oncogenic mechanisms [24, 25], SHH inhibitors were more effective than the previously tested compounds in preventing CAC through inhibition of IL-6 and CSC ablation.

Concurrent with the inhibition of IL-6 signaling, another significant effect of SHH inhibitors observed in the current study was blocking of inflammation-prone oncogenic mechanisms *via* induction of NAD-dependent 15-PGDH, which plays a major role in catabolism of the naturally occurring prostaglandin PGE_2_ by oxidizing its 15-hydroxyl group to inactive 15-keto metabolites; the induction of 15-PGDH was one of the prominent preventive effects of SHH inhibitors against CAC. 15-PGDH is highly expressed in normal colon epithelium, but its expression is strongly decreased in colon cancers [20]; therefore, agents that both enhance 15-PGDH expression and suppress COX-2 production may more effectively prevent CAC, as exemplified by the achievements with a synthetic triterpenoid or NSAIDs. We speculate that one of the core mechanisms of how SHH inhibitors prevent CAC can be explained by the finding that inflammatory cytokines (IL-6 in the current study) significantly suppress 15-PGDH expression, whereas SHH inhibitors significantly preserve 15-PGDH in the colon (Figure 6F).

In addition to the regulation of inflammatory mediators by SHH inhibitors, previous studies have shown that Bcl-2 is a target protein of *Gli* in the SHH signaling pathway [31] because *Gli* regulates the expression of Bcl-2. For instance, in a study on basal cell carcinoma, the Bcl-2 promoter was found to have seven potential *Gli* anchor points, which enable *Gli* proteins to regulate Bcl-2 activity through transcriptional regulation of the *Bcl*-2 promoter. In medulloblastoma samples, *Gli-1* expression was clearly correlated with *Bcl*-2 mRNA levels. Therefore, when cyclopamine blocks SHH signaling to decrease *Gli-1* mRNA expression, a corresponding decrease in *Bcl-2* mRNA can occur, which can promote apoptosis of tumor cells. Furthermore, an SHH inhibitor reduced viability and promoted apoptosis in the cancer cell line HCT-116. These effects were observed together with an increase in the cleaved forms of apoptotic proteins caspase-3 and PARP, and a reduction in anti-apoptotic Bcl-2 and survivin levels (Figure 3). Furthermore, an SHH inhibitor also inhibited proliferation through decreased levels of CDK4, cyclin D, and cyclin E and increased p21.

Recent publications have shown that, although HH signaling is associated with the neoplastic process, SHH is very important as an essential niche factor that maintains touch-dome stem cells [32]. On the contrary, contributing factors of CSCs, especially in cancer management, are their “bad” functions including treatment resistance, recurrence, and metastasis; usually their activation of different signaling pathways such as Notch, Wnt/β-catenin, PI3K/Akt/mTOR, JAK/STAT, and HH signaling pathways [33]. Therefore, even though there is a crosstalk between different embryonic developmental signaling pathways relevant to CSCs [34], novel chemotherapeutic agents that target CSCs (either individually or, ideally, in combinations) to inhibit compensatory escape mechanisms are attractive pharmaceutical candidates. Although our knowledge of the mechanisms of activation of the Hh pathway in CSCs from most tumor types is limited [35], several studies clearly indicate that CSCs from many tumor types are sensitive to HH inhibitors and that HH-targeted therapeutics can block transforming pathways that lead to CSCs. In the current study, we found that SHH inhibitors were very effective in either blocking tumorsphere formation or killing already formed tumorspheres (Figure 5B), leading to significant inhibition of colonsphere xenografts (Figure 7B).

In conclusion, SHH inhibition can be an ideal opportunity to seize both mutagenic inflammation and CSC features to prevent cancer, simply achieving chemoquiescence. However, further efforts to develop an efficient, potent, and safe SHH inhibitor are required for eventual clinical application.

## MATERIALS AND METHODS

### Reagents

SHH inhibitors including cerulenin, cyclopamine, and itraconazole were purchased from Sigma-Aldrich (St. Louis, MO). Recombinant human TNF-α (rhTNF-α) was purchased from R & D Systems (Minneapolis, MN). Primary antibodies for western blotting were purchased as follows: ERK, pERK, NF-kB p50, FAS, FADD, XIAP, Survivin, and β-actin from Santa Cruz Biotechnology (Santa Cruz, CA); Cox-2 was from Thermo Scientific (Fremont, CA); other antibodies from Cell signaling technology (Danvers, MA). Horseradish peroxidase-conjugated anti-rat/rabbit/mouse/goat IgG was purchased Santa Cruz Biotechnology (Santa Cruz, CA). Primers for RT-PCR were synthesized by Bioneer (Seoul, Korea). Reverse transcriptase was from Promega (Madison, WI). Azoxymethane (AOM) was purchased from Sigma-Aldrich (St. Louis, MO) and dissolved in distilled water. Dextran sulfate sodium salt (DSS) was purchased from MP biomedicals (Morgan Irvine, CA). All other materials were obtained in the highest available grade.

### Cell culture

Rat intestinal epithelial cells (IEC-6) and human colorectal cells HCT-116 were obtained from the American Type Culture Collection (ATCC, Rockville, MD) and maintained according to the ATCC's instructions. IEC-6 cells were maintained in DMEM medium and HCT-116 were maintained in McCoy's 5a Medium Modified containing 10% fetal bovine serum (FBS) (Hyclone, Thermo, Utah, USA), and 1% penicillin /streptomycin at 37°C in a humidified atmosphere with 5% CO_2_.

### Preparation of cytoplasmic and nuclear protein extract

Nuclear–cytoplasmic fractionation was conducted using the NE-PER Nuclear and Cytoplasmic Extraction Reagents kit (Thermo Fisher Scientific, Rockford, lL) according to the manufacturer's protocol. The protein concentration was measured by BCA protein assay reagent and stored extracts at −80°C until use.

### Western blot analysis

Equal amounts of protein were separated by SDS-PAGE and electrophoretically transferred to polyvinylidene fluoride (PVDF) membranes (Millipore, Bedford, MA, USA). The membrane was blocked at room temperature with 5% nonfat dry milk in PBS containing 0.1% Tween (PBS-T). Membrane was washed three with PBS-T and incubated with the primary antibodies overnight at 4°C. After washing three times with PBS-T, the membrane was incubated with the second antibodies at RT for 1 hour with 3% nonfat dry milk in PBS-T, and the membrane was washed with PBS-T for three times. The membrane was visualized using West-zol Plus (Intron biotechnology, Seongnam).

### RNA isolation and RT-PCR

Total RNA were isolated from cells using TRIzol (Life technologies, Carlsbad, CA). The cells were washed twice time with PBS and then incubated in TRIzol for 10 min at 4°C. Further 200 μL of chloroform was added and gently mixed. After incubation for 10 min on ice, samples were centrifuged at 13,200 rpm for 15 min. Supernatants were mixed with 500 μL of isopropanol, and incubated at 4°C for 15 min. After centrifuging at 13,200 rpm for 10 min, pellets were washed with 70% (v/v) ethanol. After allowing the ethanol to evaporate completely, pellets were dissolved in 100 μL of diethylenepyrocarbonate (DEPC)-treated water (Invitrogen Life Technologies, Carlsbad, CA). The quality of total RNA was measure by the ratio of A260/A280 using Nanodrop 2000 spectrophotometer (Thermo Scientific, Wilmington, DE). cDNA was prepared using reverse transcriptase originating from Murine Moloney leukemia virus (Promega, Madison, WI), according to the manufacturer's instructions. PCR was performed 30 cycles of: 94°C for 30 s, 58°C for 30 s, and 72°C for 45 s. The sequences of PCR primers used were as follows (See Supplementary Table 1).

### Levels of IL-6 by ELISA

IEC-6 cells pretreated with SHH inhibitors for 1 hour and TNF-α was then treated for 6 hours. Fresh cell culture supernatants were collected and concentrated using Amicon Ultra-15 centrifugal filter devices (Millipore, Billerica, MA). IL-6 was determined in the cell culture supernatant using ELISA kits (R & D, Mineapolis, MN) according to manufacturer's instruction. All samples were measured for their individual levels, and each sample was analyzed in triplicate manner, taking the mean of the three determinations.

### Immunofluorescence staining and confocal laser microscopy

Cells were seeded onto four-chamber slides. Slides were fixed in a 95%MeOH/5%acetic acid for 15 min. and incubated for 2 hours at room temperature with 5% bovine serum albumin (BSA) blocking buffer under shaking. Slides were rinsed once with PBS and 0.05% Tween 20 and then incubated with appropriately diluted primary antibodies in PBS. Cells were incubated with primary antibody for overnight at 4°C. After incubation, the slides were rinsed three times and incubated with FITC-conjugated secondary antibody for 1 hour at room temperature. The cells were stained with nuclear dye DAPI (Vector Labs, Inc., Burlingame, CA) and mounted with mounting medium. Expression and localization of the proteins were observed with confocal laser scanning microscope for capturing representative images.

### Transient transfection and the luciferase reporter activity assay

IEC-6 cells were seeded at a density of 1 × 10^5^ cells per well in 24-well plates and grown to 80% confluence in complete growth medium. Cells were transiently cotransfected with 0.8 μg/well of translucent IL-6 or Cox-2 reporter vector. IL-6-luciferase reporter construct were gift from Dr. Seong Ho Jeon (Hanllym University, Chooncheon, Korea) and Cox-2-luciferase reporter construct were gift from Dr. Byung Ju Lee (Ulsan University Hospital, Ulsan, Korea). After 24 hours of transfection, cells were pretreated with cerulenin or cyclopamine for 1 hour and followed by stimulating with TNF-α for 6 hours. The cell were then washed with PBS and lysed in 1x reporter lysis buffer (Promega). The lysed cell extract (20 μl) was mixed with 100 μl of luciferase assay reagent (Promega) and the luciferase activity was measured by the luminometer (MDS Analysis Tecnologies). The β-galactosidase assay was done according to the supplier's instructions (Promega β-galactosidase enzyme assay) for normalizing the luciferase activity. All data are expressed as the percentage of activity relative to basal activity.

### Transcription factor activity assay

NF-κB DNA-binding activity assays were performed using a 96-well enzyme-linked immunosorbent assay (ELISA)–based kit from Abcam (Cambridge, MA), according to the manufacturer's protocol. Briefly, NF-κB contained in a nuclear extract binds specifically to the NF-κB response element. NF-κB p50 and p65 subunit are detected by addition of a specific primary antibody directed against NF-κB p50 or p65. A secondary antibody conjugated to HRP is added to provide a sensitive colorimetric readout at 450 nm.

### Cell viability assay

HCT-116 cells were seeded at a density 1 × 10^4^ cells/well in 24-well plate. After overnight attachment, cells were incubated with different concentration of cerulenin and cyclopamine for 24 hour. The cell viability was determined using the 3-(4,5-dimethylthiazol-2-yl)-2,5-diphenyltetrazolium bromide (MTT) (Sigma-Aldrich, St. Louis, MO, USA) reduction assay. After incubation for different concentrations of drugs, cells were treated with the MTT solution (final concentration, 1 mg/ml) at 37°C for 4 hour. The dark blue formazan crystals formed in intact cells were dissolved with equal amounts of dimethyl sulfoxide (DMSO) and the absorbance at 570 nm was read using a microplate reader. Results were expressed as the fold increase of control.

### Assessment of apoptosis with Annexin-V assay

HCT-116 cells were plated at a density of 1.5 × 10^5^ cells/mL. After overnight attachment, cells were treated with cerulenin induce apoptosis in triplicate, for 24 hr, followed by washing with PBS, trypsinization, and centrifugation. Cells were stained with the Annexin-V kit (BD Biosciences, San Jose, CA). Briefly, cells were washed twice in cold PBS, resuspended in binding buffer and 10 ml of FITC-conjugated Annexin-V were added. Cells were incubated for 15 min in the dark, an additional 400 mL of binding buffer were added, and the cells were analyzed within 1 hour by flow cytometry. Acquisition was performed on a FACSCalibur using the CellQuest Pro software (BD Biosciences, San Jose, CA), each analysis was performed on at least 30,000 events.

### 5-bromo-2′-deoxyuridine (BrdU) incorporation assay

Cells were seeded and serum starved. After treatment with PBS or SHH inhibitors at different concentrations for 24 hours in 10% FBS-containing media, cells were incubated with BrdU for an additional 2 hours. BrdU-positive cells were counted under a light microscope.

### Wound healing assay

HCT-116 cells (2 × 10^5^) were seeded in 6-well plates. When 80% confluence of cell growth was achieved, cells were scraped across the cell monolayer using a 200 μl plastic tip. Photomicrographs were captured at 0, 24 hours. Results were expressed as the migration index (%). The experiment was repeated 3 times, independently.

### Terminal deoxynucleotidyl transferase-mediated dUTP nick end labeling assay (TUNEL)

TUNEL assay kit (Promega, Madison, WI) was performed to verify apoptosis according to manufacturer's instruction. Briefly, after dewaxing and rehydrating, the sections were incubated first with proteinase K (20 μg/ml) for 10 min at room temperature, then with TdT and fluorescein-12-dUTP for 1 hour at 37°C, and finally stain with DAPI for 5 min at RT. The samples were mounted and TUNEL-positive cells were counted under a fluoresce microscopy.

### RayBio apoptosis array

A human apoptosis antibody array kit (Ray Biotech, Norcross, GA) was used to detect the relative levels of apoptosis-related proteins in the cell lysates according to the manufacturer's instructions. Briefly, the treated cell lysates were added to the antibody array membranes and incubated overnight with rocking at 4°C. After washing membrane, the membranes were incubated with a cocktail of biotin-conjugated anti-antibodies to apoptotic proteins at room temperature for 2 hours. After incubation with horseradish peroxidase–streptavidin at room temperature for 1.5 hours, the signals were visualized by chemiluminescence.

### Tumorspheres culture and colosphere formation assay

Tumorspheres were generated by seeding the HCT116 adherent cells into serum-free DMEM culture medium containing, B27 (1x, Gibco), EGF (20 ng/ml, Peprotech), bFGF (10 ng/ml, Peprotech), 1% antibiotic mixture (invitrogen), and the healthy adherent cells were plated in 100-mm ultra-low attachment plate (Corning) at 1 × 10^6^ cells per plate in humidified incubator at 37°C in 5% CO2. After 7 days, the primary tumorspheres were counted and dissociated at the density of 1,000 cells per milliliter and 500 mL single cell suspension was seeded in each well of 24-well ultra-low attachment plate (Corning) in serum-free medium described above. After 7 days later, secondary tumorsphere were counted and took picture under inverted microscopy.

### Soft agar colony formation assay

To examine the self-renewing capacity, each well of a six-well plate was coated with 1 mL of 10% FBS DMEM medium with 0.6% agarose. After 30 min of incubation at 37°C, equal numbers (10^4^) of tumorsphere cells or adherent cells were added in 1 mL of 10% FBS 1640 medium with 0.3% agarose. After treatment SHH inhibitors, plates were incubated under standard conditions and with the addition of 300 mL of medium every 3 days for 3 weeks. Colonies were stained with 0.01% crystal violet and counted colonies under a light microscope.

### Animal study and treatment

Specific pathogen free male C57BL/6 mice (Six weeks old) were purchased from Orient Bio Inc. (Seongnam, Korea). All mice were divided into 5 experimental groups (10 mice per group in random). For assessment ACF formation, the mice received a single intraperitoneal injection of AOM at a dose of 3 mg/kg body weight. Starting 1 week after the AOM injection, animals were exposed to 2% DSS in the drinking water for 7 days. Cerulenin (10 or 30 mg/kg/day), Itraconazole (100 mg/kg/day) were given orally daily for 2 weeks after AOM injection 7 days. Mice were then sacrificed at the end of the 3rd week. The other groups for assessment tumor development, the mice were maintained with SHH inhibitors treatment until the 16th week. These mice were given orally 3 times per week of cerulenin (1 or 10 mg/kg) or itraconazole (100 mg/kg) after AOM injection 7 days. All animals were killed by CO_2_ asphyxiation. After laparotomy, the lengths of colon were measured, and isolated tissues were subjected to a histologic examination and extraction of protein. Animals were handled in an accredited animal facility in accordance with Association for Assessment and Accreditation of Laboratory Animal Care International (AAALAC International) guidelines under the facility named CACU (The Center of Animal Care and Use) of CHA University Laboratory Animal Research Center after IRB approval.

### Histopathology assessment and immunohistochemical staining

Isolated colon tissues for histologic examination were spread onto a plastic sheet, fixed in 3.7% formalin for 24 hours, and prepared for paraffin tissue slides. The paraffin sections were stained with hematoxylin and eosin (H & E) or saved for immunohistochemical staining. Briefly, after dewaxing and rehydrating, antigen retrieval was performed by boiling in 10 mM sodium citrate buffer, pH 6.0. Endogenous peroxidase was quenched using 3% hydrogen peroxide in methanol for 20 min. Sections were incubated with mouse antibodies F4/80 (1:500, eBioscience, San Diego, CA), mouse NF-κB p65 (Cell signaling technology, Danvers, MA), overnight at 4°C. The sections were incubated with a biotinylated secondary antibody and subsequently incubated in VECTORSTAIN ELITE ABC reagent for 30 min, using 3,3′-diaminobenzidine (Vector Laboratories, Burlingame, CA) as the chromogen. Sections were then counterstained for 1–2 min with hematoxylin (Sigma, St Louis, MO) and mounted with Permount. Scoring was estimated as the percentage of positively stained cells of whole tissue and performed by the first author, who was blinded as to the primary antibodies and the treatment groups.

### Statistical analysis

All data are presented as the means ± SD of three independent experiments. The data were analyzed by one way analysis of variance (ANOVA) test. The Fisher post hoc test was use for significance of difference between experimental groups. Differences were considered statistically significant at *P* value < 0.05.

## SUPPLEMENTARY FIGURES AND TABLE


