# Supplementary material for: Sonic hedgehog inhibitors prevent colitis-associated cancer via orchestrated mechanisms of IL-6/gp130 inhibition, 15-PGDH induction, Bcl-2 abrogation, and tumorsphere inhibition
Source: Oncotarget. 2015 Dec 26;7(7):7667–82. doi: 10.18632/oncotarget.6765 (PMC4884946; doi:10.18632/oncotarget.6765)
Supplement: Supplementary file 1 [file oncotarget-07-7667-s001.pdf]

Sonic hedgehog inhibitors prevent colitis-associated cancer via orchestrated mechanisms of IL-6/gp130 inhibition, 15-PGDH induction, Bcl-2 abrogation, and tumorsphere inhibition

Supplementary Materials

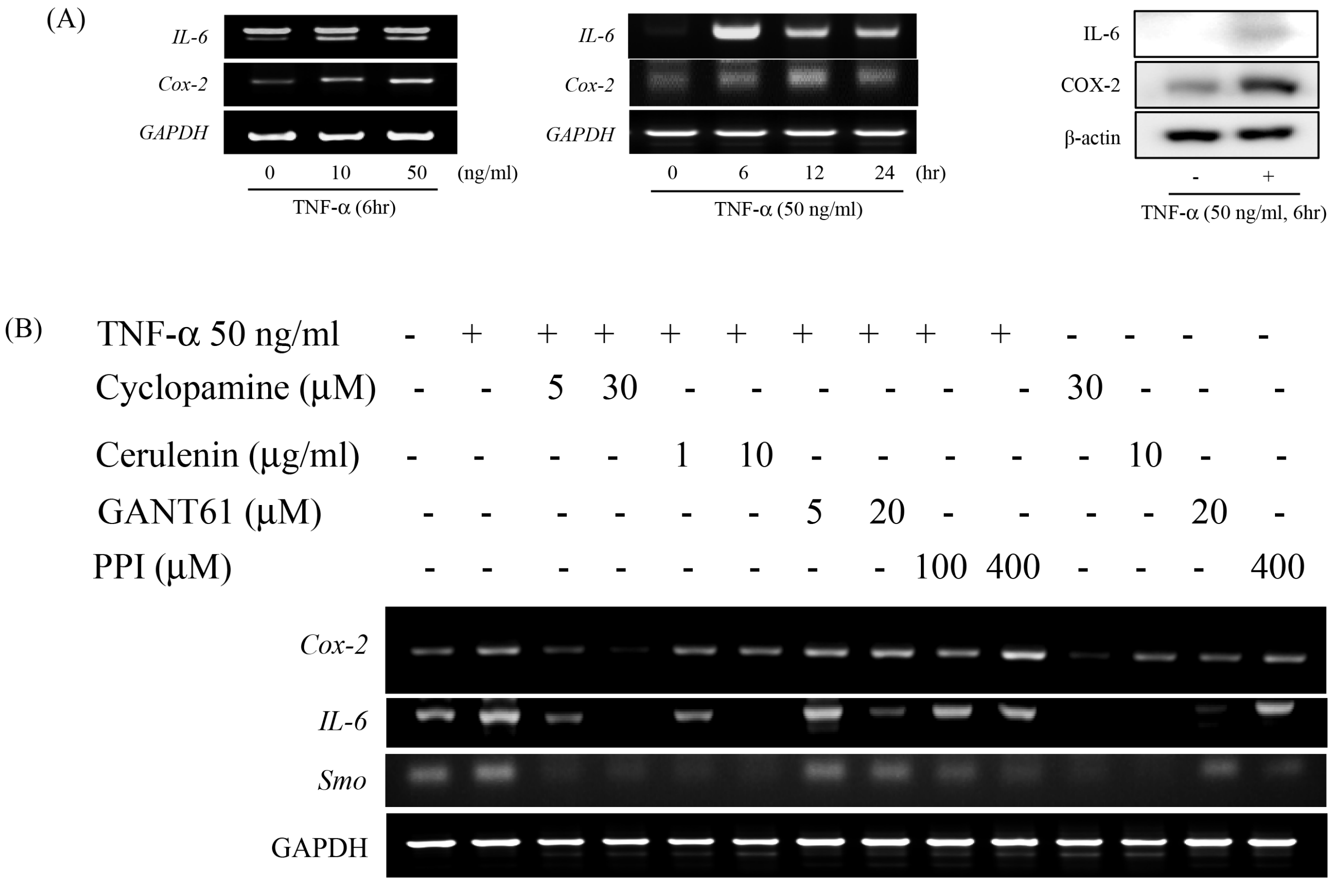

Supplementary Figure S1: (**A**) Changes in IL-6 and *Cox*-2 after treatment with different doses of TNF-α: stimulation with 50 ng/ml TNF-α for 16 h significantly induced IL-6 and COX-2, after which whole *in vitro* study was challenged with 50 ng/ml TNF-α for 16 h in an *in vitro* model. (**B**) Changes in *Cox-2, IL-6*, and *Smo* mRNAs after treatment with different doses of SHH inhibitors.

(A)

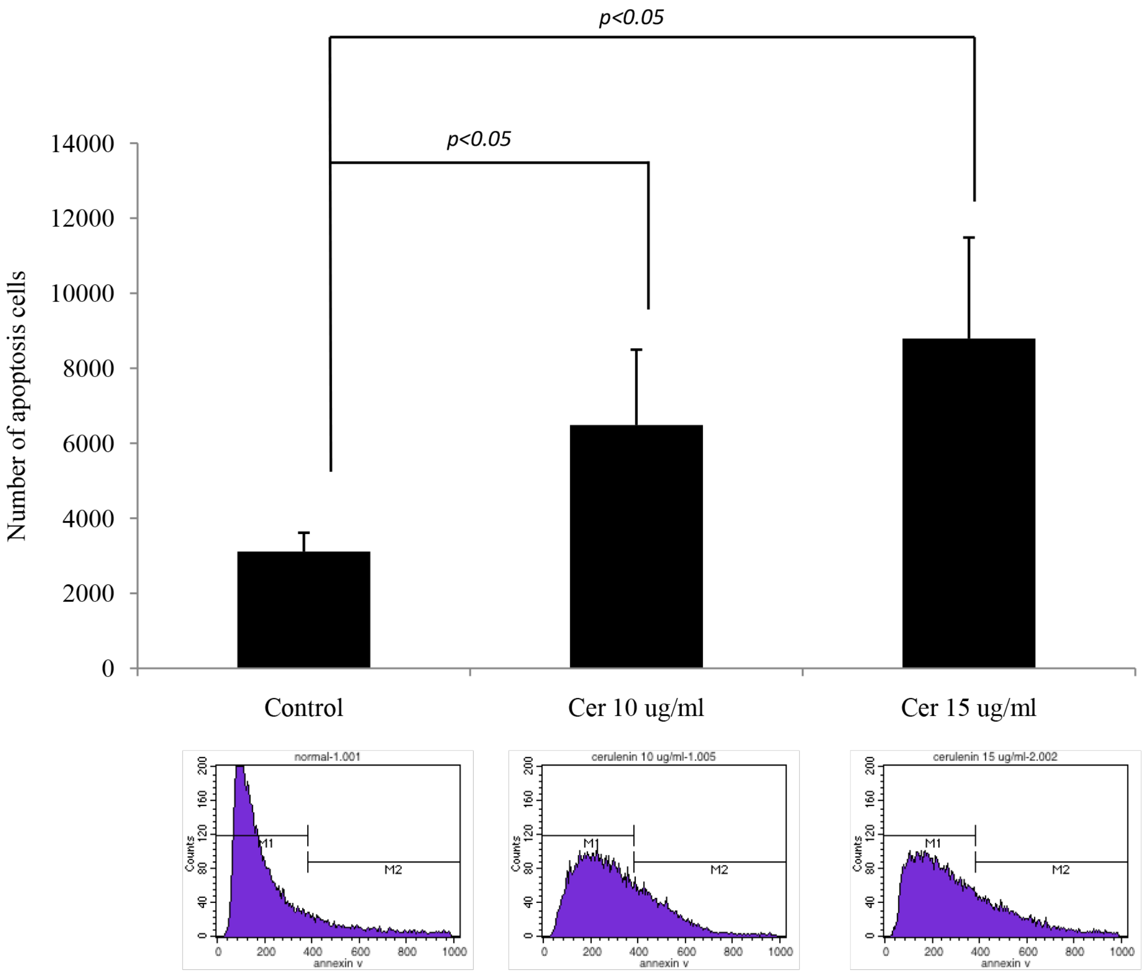

(B)

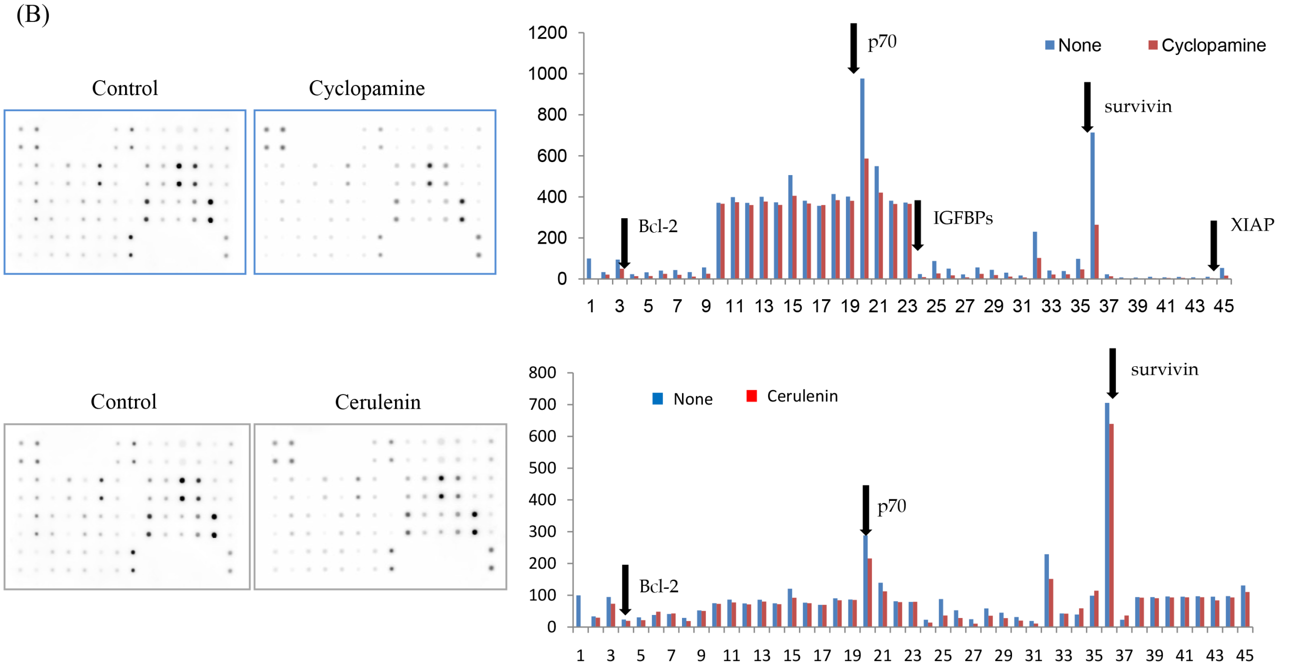

1	Positive	6	BID	11	CD40L	16	FasL	21	HTRA	26	IGFBP-3	31	livin	36	Survivin	41	TRAILR-1
2	bad	7	BIM	12	cIAP-2	17	blank	22	IGF-I	27	IGFBP-4	32	p21	37	sTNF-R1	42	TRAILR-2
3	bax	8	Caspase 3	13	Cyto C	18	HSP27	23	IGF-II	28	IGFBP-5	33	p27	38	sTNF-R2	43	TRAILR-3
4	Bcl-2	9	Caspase 8	14	DR6	19	HSP60HS	24	IGFBP-1	29	IGFBP-6	34	p53	39	TNF-alpha	44	TRAILR-4
5	Bcl-w	10	CD40	15	Fas	20	P70	25	IGFBP-2	30	IGF-1sR	35	SMAC	40	TNF-beta	45	XIAP

(C)

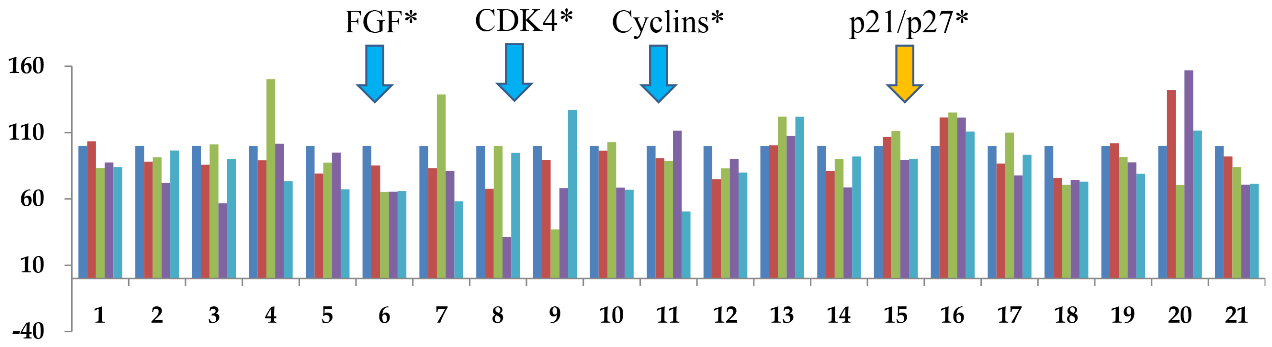

1	Ptch1	6	bFGF*	11	cyclinD1*	16	P27*	21	IGF-1
2	Shh	7	CDK1	12	cyclinE1*	17	P53		
3	Smo	8	CDK4*	13	ICAM-1	18	VCAM-1		Increased
4	VEGF	9	CDK6	14	P16	19	Bax		Decreased
5	aFGF	10	cyclinB*	15	P21*	20	Bcl-2		No meaning

A none
B cyclopamine (25μM)
C cyclopamine (50μM)
D cerulenin (25μg/ml)
E cerulenin (50μg/ml)

* *p<0.01* (vs A group, non-treated)

(D)

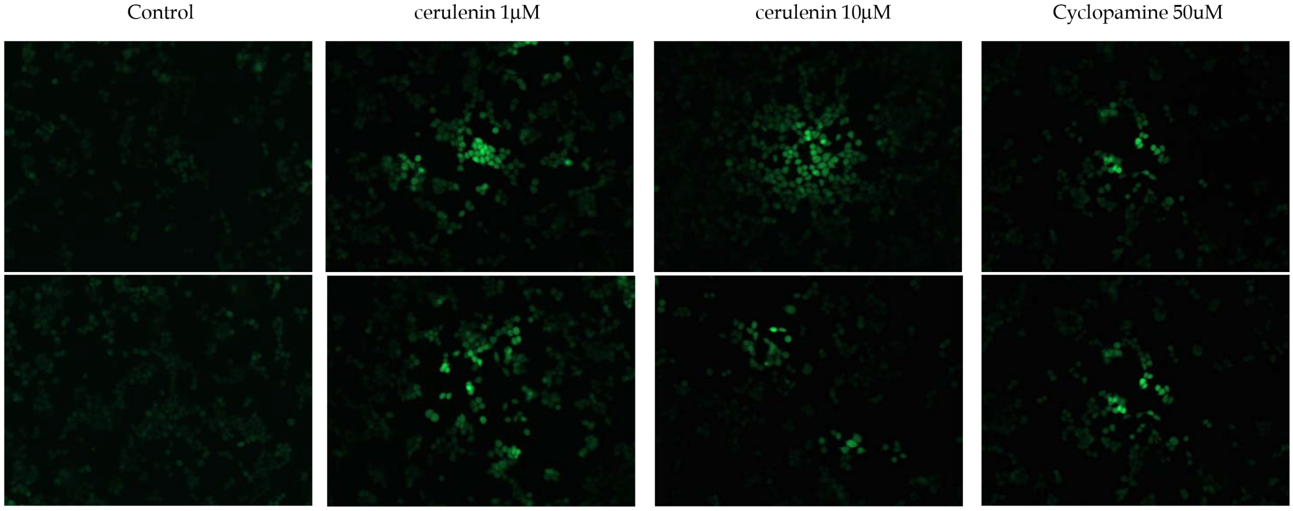

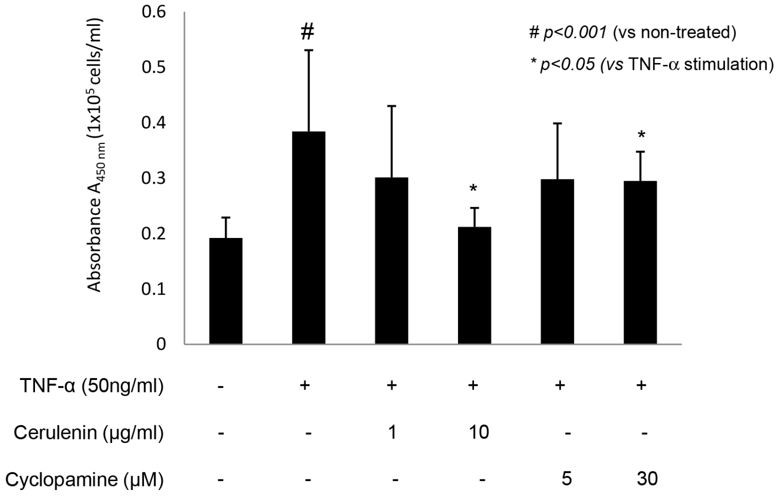

Supplementary Figure S2: (**A**) Flow cytometry for apoptosis after treatment with 10 or 15 mg/ml cerulenin. (**B**) Changes in the levels of 44 apoptosis-related proteins were measured after treatment with an SHH inhibitor (cyclopamine or cerulenin) using a protein array. (**C**) Multiplex qRT-PCR was performed to check the changes in the expression of cell cycle–related genes after treatment with SHH inhibitors. **p* < 0.01 (*vs.* untreated). (**D**) Confocal imaging of DCFH-DA after treatment with an SHH inhibitor (upper part) and the mean changes in fluorescence (lower part).

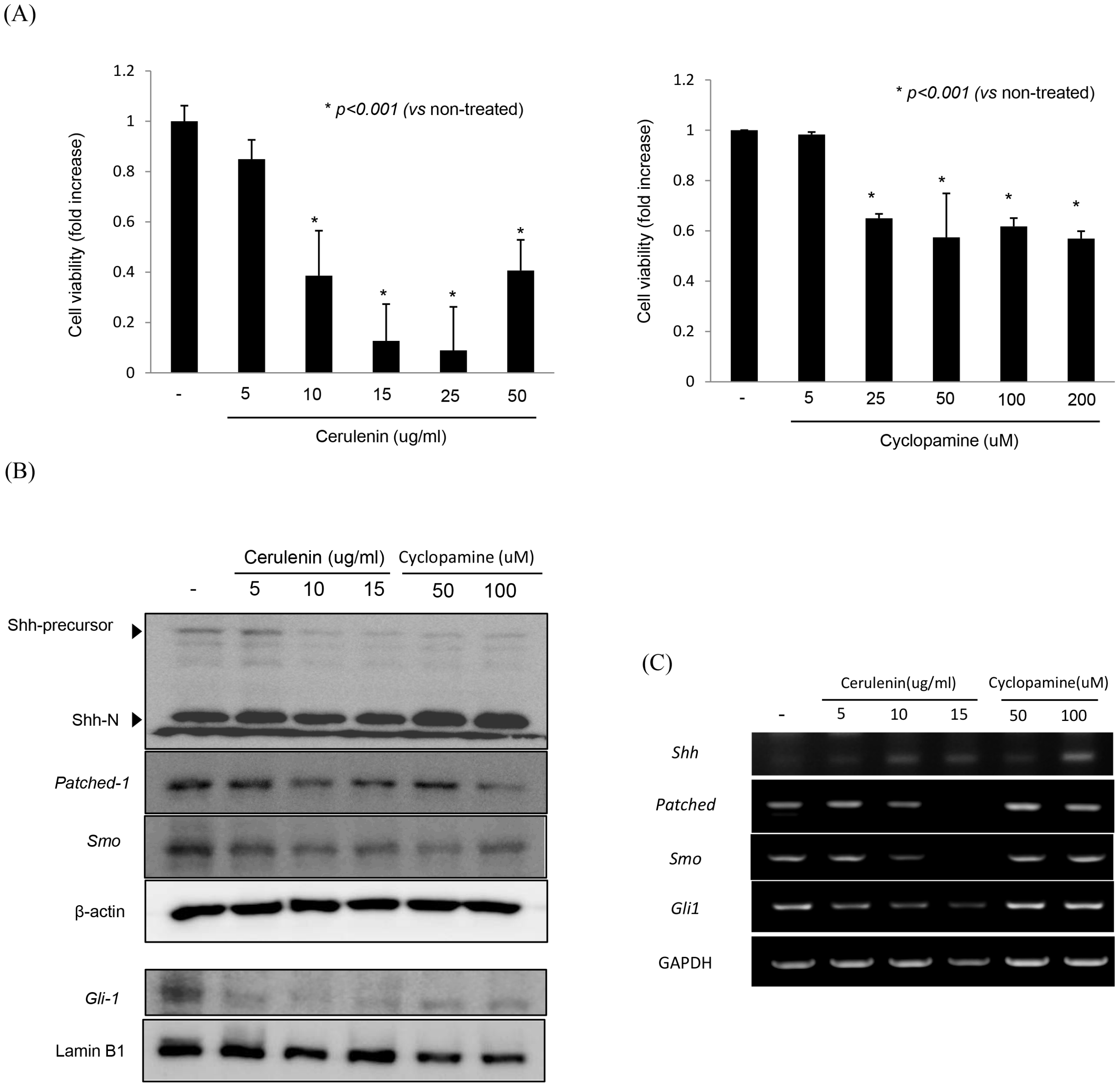

Supplementary Figure S3: (**A**) HT-29 cells were treated with the indicated concentrations of SHH inhibitors for 24 h, and their viability was determined by the MTT assay. The results are presented relative to the viability of untreated cells (control), which was considered as 1, and are means ± SD. *p < 0.01 in comparison with the untreated group. (**B**) The expression of SHH pathway proteins including Shh, Patched-1, Smo and Gli-1 was assessed by Western blot analysis after treatment with an SHH inhibitor. (**C**) Changes in the levels of *Shh, Patched, Smo*, and *Gli-1* mRNAs in the presence of different doses of SHH inhibitors. All data are representative of at least three independent experiments and are mean ± SD; *p < 0.05, **p < 0.01 in comparison with the control.

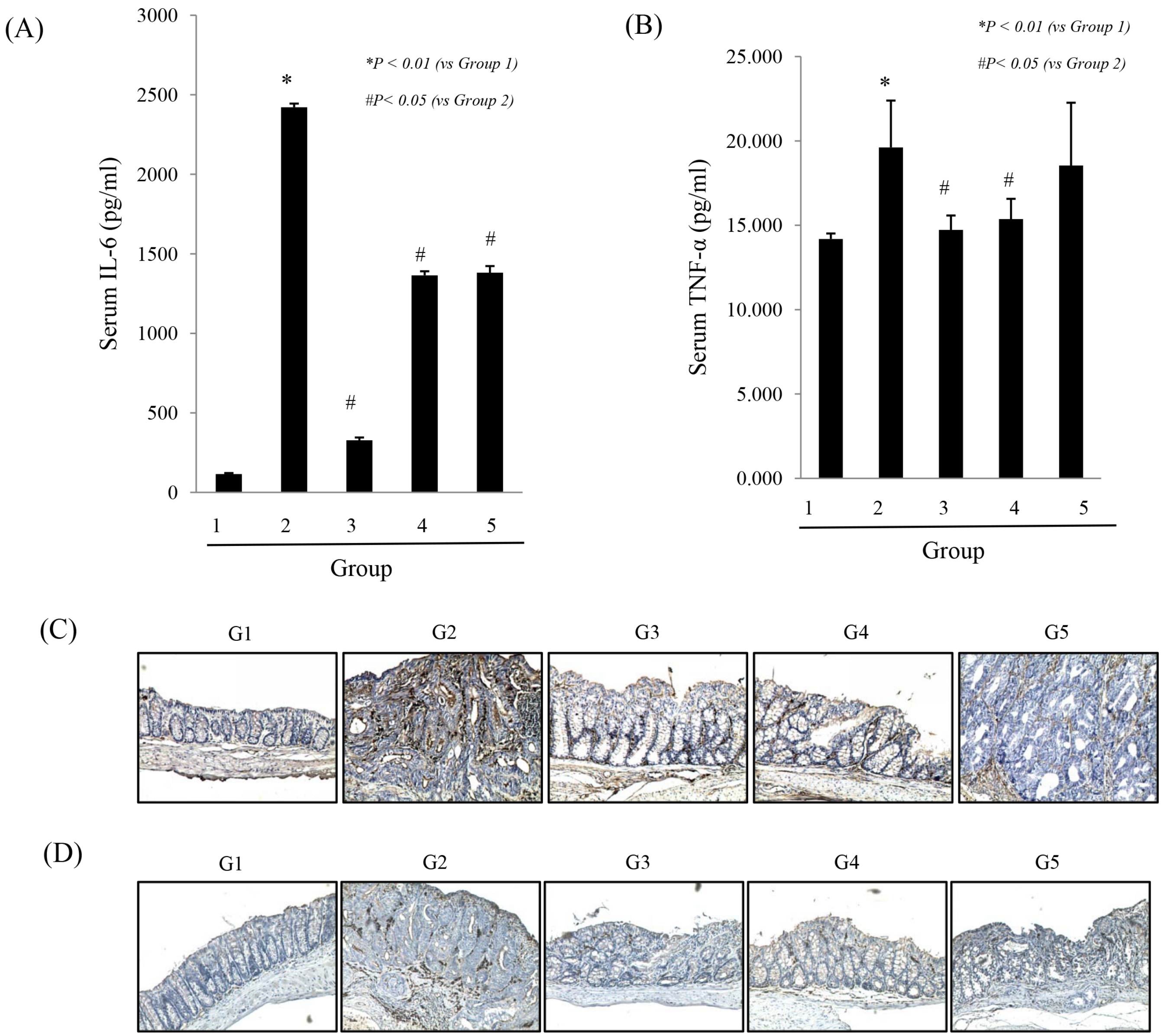

Supplementary Figure S4: (**A**) Changes in the mean serum levels of IL-6 in different groups. *$p < 0.01$ (*vs*. Group 1); $^{\#}p < 0.05$ (*vs*. Group 2). (**B**) Changes in the mean serum levels of TNF-α in different groups. *$p < 0.05$ (*vs*. Group 1); $^{\#}p < 0.05$ (*vs*. Group 2). (**C**) Representative immunohistochemical staining for NF-κB p65 in different groups. (**D**) Representative immunohistochemical staining for F4/80 in different groups.

Supplementary Table S1: Primer sequences

Primers	Species	Forward (5′–3′)	Reverse (5′–3′)
IL-6	Rat	AAG AGA CTT CCA GCC AGT TG	TGG ATG GTC TTG GTC CTT AG
IL-6R	Rat	TCA CAG AGC AGA GAA TGG ACT	GTA TGG CTG ATA CCA CAA GCT
gp130	Rat	TCA ACT TGT GGA ACC ATG TGG	TCC AAC TGA CAC AGC ATG TTC
SOCS3	Rat	CAG CTC CAA GAG CGA GTA CCA G	CAT GTA GTG GTG CAC CAA CTT GA
Grb2	Rat	GCG AGA GCG CTC CTG GGG AT	TTG CGG GGA AAC ATG CCG GT
Gab1	Rat	CAG CAG CTT CGC CGA ACC GA	TGC TGC TGC CCG AAC CAC TG
IL-11	Rat	TTG GCC ATG AGC GCT GGG AC	TGG GGC AAG GCT AGG CGA GA
IL-11RA	Rat	GGC CAT GCC CAC AAG ACC CC	GCC CAG CCA CGG CAT CTG TT
Cox-2	Rat	GAA ATG GCT GCA GAG TTG AA	TCA TCT AGT CTG GAG TGG GA
TNF-α	Rat	TAC TGA ACT TCG GGG TGA TT	CAG CCT TCT CCC TTG AAG AG
iNOS	Rat	TTT TCC CAG GCA ACC AGA CG	GTA GCG GGG CTT CAG AAT GG
IL-8	Rat	CAG ACA GTG GCA GGG ATT CA	TTG GGG ACA CCC TTT AGC AT
GAPDH	Rat	GGT GCT GAG TAT GTC GTG GA	TTC AGC TCT GGG ATG ACC TT
CDK4	human	CTG AGA ATG GCT ACC TCT CGA TAT G	AGA GTG TAA CAA CCA CGG GTG TAA G
CDK6	human	CCG AGT AGT GCA TCG CGA TCT AA	CTT TGC CTA GTT CAT CGA TAT C
Cyclin D	human	CTG TGC TGC GAA GTG GAA ACC AT	TTC ATG GCC AGC GGG AAG ACC TC
Cyclin E	human	GAA GGC CCT TAA GTG GCG TTT	TGC GGC AGT AGC ACT TCA TGT
GAPDH	human	AGG TCG GAG TCA ACG GAT TTG G	ACA GTC TTC TGG GTG GCA GTG ATG
IL-6	mouse	CCG GAG AGG AGA CTT CAC AG	TGG TCT TGG TCC TTA GCC AC
TNF-α	mouse	ATG AGC ACA GAA AGC ATG ATC	TAC AGG CTT GTC ACT CGA ATT
GAPDH	mouse	AAT GTA TCC GTT GTG GAT CT	TCC ACC ACC CTG TTG CTG TA
